# Genetic versus Rearing-Environment Effects on Phenotype: Hatchery and Natural Rearing Effects on Hatchery- and Wild-Born Coho Salmon

**DOI:** 10.1371/journal.pone.0012261

**Published:** 2010-08-19

**Authors:** Cedar M. Chittenden, Carlo A. Biagi, Jan Grimsrud Davidsen, Anette Grimsrud Davidsen, Hidehiro Kondo, Allison McKnight, Ole-Petter Pedersen, Peter A. Raven, Audun H. Rikardsen, J. Mark Shrimpton, Brett Zuehlke, R. Scott McKinley, Robert H. Devlin

**Affiliations:** 1 Centre for Aquaculture and Environmental Research, The University of British Columbia and Department of Fisheries and Oceans, West Vancouver, British Columbia, Canada; 2 Department of Arctic and Marine Biology, University of Tromsø, Tromsø, Norway; 3 Laboratory of Genome Science, Tokyo University of Marine Science and Technology, Tokyo, Japan; 4 University of Northern British Columbia, Prince George, British Columbia, Canada; Lund University, Sweden

## Abstract

With the current trends in climate and fisheries, well-designed mitigative strategies for conserving fish stocks may become increasingly necessary. The poor post-release survival of hatchery-reared Pacific salmon indicates that salmon enhancement programs require assessment. The objective of this study was to determine the relative roles that genotype and rearing environment play in the phenotypic expression of young salmon, including their survival, growth, physiology, swimming endurance, predator avoidance and migratory behaviour. Wild- and hatchery-born coho salmon adults (*Oncorhynchus kisutch*) returning to the Chehalis River in British Columbia, Canada, were crossed to create pure hatchery, pure wild, and hybrid offspring. A proportion of the progeny from each cross was reared in a traditional hatchery environment, whereas the remaining fry were reared naturally in a contained side channel. The resulting phenotypic differences between replicates, between rearing environments, and between cross types were compared. While there were few phenotypic differences noted between genetic groups reared in the same habitat, rearing environment played a significant role in smolt size, survival, swimming endurance, predator avoidance and migratory behaviour. The lack of any observed genetic differences between wild- and hatchery-born salmon may be due to the long-term mixing of these genotypes from hatchery introgression into wild populations, or conversely, due to strong selection in nature—capable of maintaining highly fit genotypes whether or not fish have experienced part of their life history under cultured conditions.

## Introduction

Climate change, over-fishing and habitat alteration are suspected to be contributing to declines in Pacific salmon stocks to the point that some populations are now seriously threatened [1]. Over the past 50 years, governments around the North Pacific, including Japan, Russia, Canada and the United States, have implemented salmon enhancement programs to increase the numbers of juvenile salmon released to oceanic conditions. These programs typically utilise the artificial propagation of returning mature adult salmon, rearing juveniles in freshwater culture conditions and releasing them as smolts. However, despite the annual release of billions of hatchery-reared fish into the Pacific Ocean, the marine survival rates of many salmon populations continue to decline [2]–[4], and the effects of these introductions on wild populations are only beginning to be understood [5], [6].

**Figure 1:**
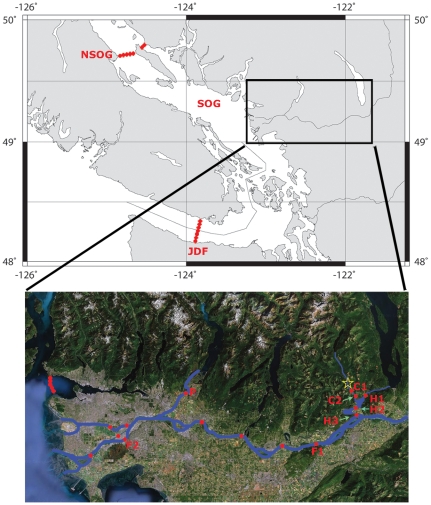
Geographic location of the study area. The Strait of Georgia (SOG), Northern Strait of Georgia receiver array (NSOG), and the Juan de Fuca Strait receiver array (JDF) are shown on the main map. The inset map displays the Chehalis River Hatchery and release site (star), the Chehalis River receivers (C1, C2), the Harrison River receiver arrays (H1, H2, H3), the Fraser River arrays (F1, F2, red dots), and the Pitt River array (P).

Concern that hatchery-reared coho salmon (coho; *Oncorhynchus kisutch*) were supplanting wild coho [7], [8] hit a peak during 2001, when 70% of the coho caught in the Strait of Georgia (SOG, Fig. 1) were reported to be of hatchery origin [9]. However, by 2006 and despite higher releases of hatchery fish from rivers in the area, the percentage of hatchery fish caught in the Strait of Georgia had dropped significantly [4]. Many factors could have been influencing this dramatic difference in survival between hatchery and wild populations. While hatcheries are highly efficient at producing large numbers of smolts under culture conditions, the physiology and behaviour of hatchery-reared smolts has been found to differ from the wild populations in many cases—e.g. [10]–[16]. The causes and effects of these differences are unclear; i.e. what are the relative roles of genotype and rearing environment on the observed phenotypic differences between wild and hatchery-reared salmonids?

Salmon are capable of a high degree of phenotypic plasticity, which means that they can physiologically and behaviourally adapt to their environment [17]. Therefore, the environment in which a young salmon is reared can have a major effect on its physiology, behaviour and survival. Similarly, significant capacity for genetic change exists within salmonid populations in response to selective pressures. For example, sockeye salmon (*O. nerka*) have been shown to evolve reproductive isolation in fewer than thirteen generations [18]. Genetic changes arising from salmon enhancement programs are currently not well understood, and may not be as evident as the effects of rearing environment in the short term, but recent studies have found that captive breeding can significantly decrease the fitness of steelhead trout (*O. mykiss*) in just one or two generations [19]. Furthermore, the carry-over effects of captive breeding into subsequent wild-born generations have been observed in the reduced fitness of some stocks [20].

The relative effects of rearing-environment and genotype on salmon phenotype, behaviour and survival are generally unknown and need to be assessed [21]. Enhanced systems can have all of the fish in the population propagated by human intervention, or they can have a mix of wild- and artificially-bred fish. Thus, in many cases there may not be any completely wild-type fish available to assess hatchery propagation effects. However, in systems where all hatchery fish are marked prior to release, it is possible to distinguish between fish that have been propagated by hatchery production from those that have lived their entire lives in nature and have arisen from parents which mated in the wild. Comparing these two types of fish allows for an assessment of the single-generation effects of hatcheries. Understanding such effects is increasingly important given future uncertainties for salmon populations (e.g. arising from climate trends), as mitigative approaches, such as enhancement programs, have the potential to alleviate some of these pressures. This study assessed the relative roles that genetics and rearing environment play in the phenotypic expression of coho young, including their growth, physiology, survival, swimming and predator-avoidance abilities, and their migratory behaviour.

## Methods

All work involving live fish reported in this paper was annually reviewed and pre-approved as meeting or exceeding the standards laid out by the Canadian Council on Animal Care. The project guidelines were approved by The University of British Columbia's Committee on Animal Care at Suite 102, 6190 Agronomy Road, Vancouver, BC (permit A06-0153).

**Figure 2:**
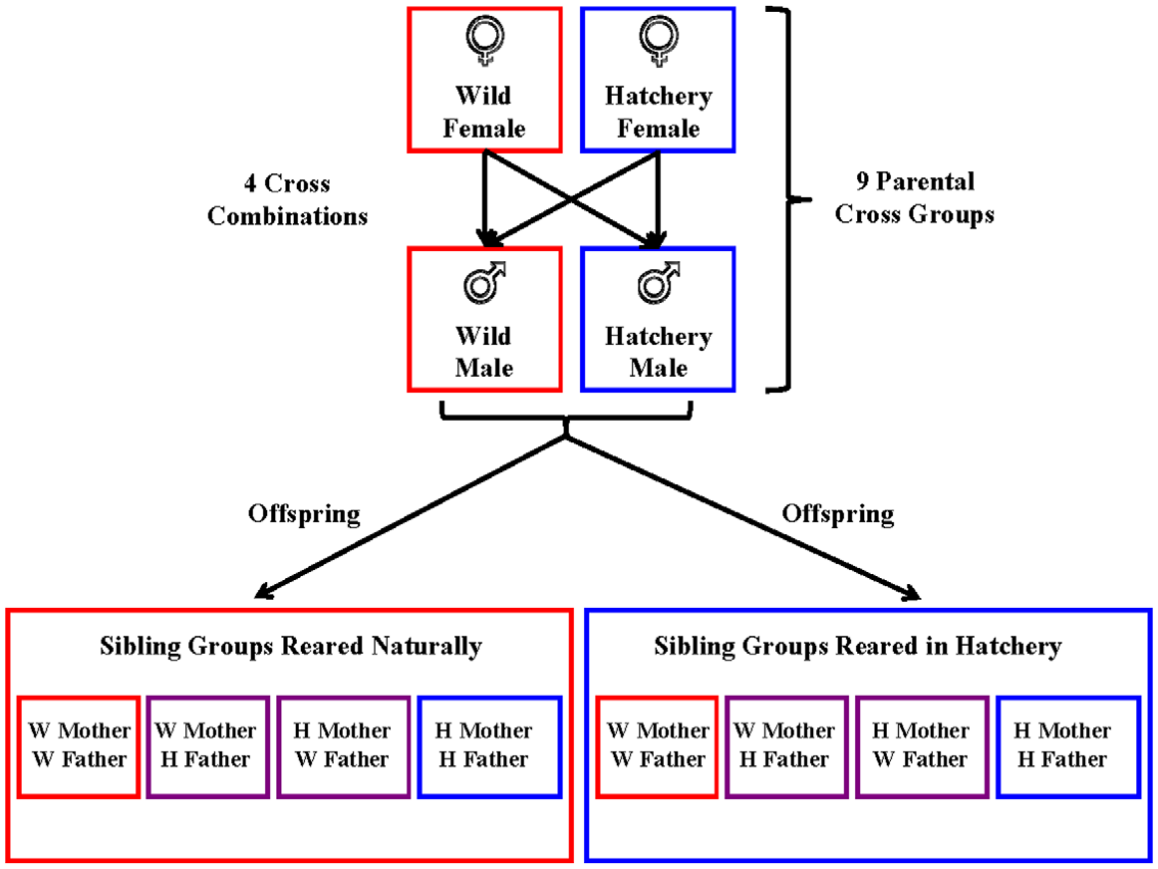
The experimental design used to determine genetic versus environmental effects on the phenotypic characteristics and fitness of wild- and hatchery-born salmon. The pure wild, pure hatchery and hybrid offspring of nine parental cross groups were included in this study. Full-sibling groups from each cross were reared in both a traditional hatchery and a natural side-channel environment.

Returning wild- and hatchery-born coho adults were crossed at the Chehalis Hatchery (Fig. 1) during the winter of 2006/2007 to create pure-hatchery-genotype, pure-wild-genotype and hybrid-genotype offspring (Fig. 2). Wild-born adults were those individuals having survived for at least one generation in the natural environment—distinguishable from hatchery fish by the lack of an adipose-fin clip. To test for genetic effects, environment effects, and genotype-by-environment-interaction effects on the smolts, one-half of the progeny from each cross group was reared in a traditional hatchery environment. The remaining half was reared in a natural side channel of the Chehalis River (Fig. S1). There were two replicates set up in each rearing environment. The following spring (May 2008), after one year in freshwater, the coho pre-smolts were recaptured for sampling (mass, length and DNA from the adipose fin). Relative survival and phenotype comparisons were made between replicate, rearing-environment, and genotype groups. The phenotypic characteristics measured included length, mass, condition factor (mass · length^−3^), survival, colour, fin quality, disease presence, gill Na^+^/K^+^−ATPase activity level, microarray gene expression profile, predator avoidance ability, swimming endurance and migratory behaviour—including speed, timing and habitat use. See *Physiology* for further details on the methods used.

Eighty crosses of returning coho adults were carried out at the Chehalis Hatchery on 1 February 2007 (n = 40 adipose-clipped/hatchery-derived, 40 unclipped/likely wild-born with either hatchery or wild parents). Blood samples were taken and stored in 0.01 N sodium hydroxide for later genetic identification. The eggs of each female parent were divided in two equal groups so that one half could be fertilized by a wild male and the other by a hatchery male to test for maternal effects (Fig. 2). Each cross group included one hatchery female, one hatchery male, one wild female and one wild male, to generate four separate crosses—one pure wild strain, one pure hatchery strain, one hybrid strain with a hatchery mother and wild father, and one hybrid strain with a wild mother and a hatchery father (Fig. 2). Nine of these cross groups were selected for the experiment (18 male and 18 female parents). Thus, in total there were 36 full-sibling egg groups that were weighed, counted, and reared separately in randomised incubation trays.

As there was a small percentage (∼10%) of the returning adults of early 2007 that were released from the hatchery unclipped, the parental otoliths were analysed to confirm which of the unclipped fish were wild-born, and which had actually been hatchery-reared. One year later (January 2008) otoliths were collected from 20 clipped and 20 unclipped returning adults from the 2004 broodstock for analysis. All of the hatchery fish from the 2004 broodstock had been adipose-clipped prior to release; therefore their otoliths could be used as a control to compare with the parental otoliths. Many of the otoliths from hatchery-origin fish were too crystallized to be able to see growth rings, so the degree of crystallization was used as an indicator of origin [22], [23]. The otoliths were examined under a microscope and given a score out of 4 for degree of crystallization (1 = 25%, 2 = 50%, 3 = 75%, 4 = 100% crystallized). Otoliths from the smolts reared naturally (n = 30) and those reared in the hatchery environment (n = 30) were also sampled for comparison (during June 2008).

### Rearing Environments

Three months post-fertilization (early May 2007), the unfed fry from each full-sibling group were divided into four rearing groups and released into the four separate habitats (two replicates in the hatchery and two in the contained natural side-channel). The two hatchery rearing groups had 100 individuals from each full-sibling group, as they had a much greater expected survival rate than those reared naturally. The natural-rearing groups had one-half of the remaining individuals from each sibling group. A total of 3,600 fry were released into each of the hatchery troughs, and 23,000 fry were released into each of the natural habitats. Whereas the coho fry in the side-channel rearing areas were left to feed on naturally-occurring food sources, the hatchery-reared fry were fed daily on a typical hatchery diet (from ponding to release: Ewos #0, #1, BioClark #1, #2, BioVita #1.2, ProForm 1 mm, 1.5 mm). To reduce the predation pressure in the natural habitats and ensure that some fry survived the year, 135 resident coho smolts and 2 cutthroat trout (*O. clarkii*) were trapped in the natural habitat areas and released into the wild. Following this removal, there were still mammalian, avian and aquatic predators observed in both of the natural habitat areas. Further detail on the natural rearing environment can be found in (File S1).

The starting density in the natural rearing area was 46,000 fish in 3,230 m^3^, or 14 fry per m^3^. The hatchery rearing troughs were uniform in size, structure and water quality. Each contained a volume of 0.9 m^3^ (3.2 m long×0.9 m wide×0.3 m deep). Thus, with 3,600 fry in each, the hatchery-rearing density was 4,167 fry per m^3^. Two months prior to their release, the pre-smolts in the hatchery were moved to a larger trough with a gate at one end connecting to the Chehalis River.

### Physiology

Size sampling was conducted monthly in the hatchery from April 2007 (fry ponding) to June 2008 (smolt release). The naturally-reared fish were trapped using baited minnow traps and sampled during February, April, May and June 2008. All of the fish were returned to their habitat following measurement. During May and June 2008, DNA samples were taken non-lethally (adipose clip) from the naturally- and hatchery-reared pre-smolts (n = 428 natural, 608 hatchery) to identify genetic cross groups. Microsatellite analysis was conducted on eight loci (Ots10, Omm1270-6, Omm1231, Omm1128, Omm5030, Omm5007, Omm5008 and OneU3) according to methods in [24]. The results were processed by a maximum likelihood program that determines full- and half-sibling groups based on genotypes [25]. Both the allele dropout rates and other typing error rates were set at 0.1 for all loci and the inferred sibling genotypes were selected only if their probability was greater than 0.98.

At the time of the smolt release during late June 2008, a physical assessment was conducted on 30 hatchery- and 30 naturally-reared fish. The smolts were euthanised prior to sampling in buffered tricaine methanesulphonate (200 ppm TMS; 400 ppm sodium bicarbonate). Mass and fork length was measured and condition factors calculated (mass · fork length^−3^) [26]. Blood was taken from the caudal vessels with a sterile syringe rinsed with lithium heparin. Gill samples were stored in a −80°C freezer until Na^+^/K+−ATPase activity assays could be conducted [27]. Organ tissue samples were also taken to test for Bacterial Kidney Disease (BKD) and Infectious Hematopoietic Necrosis (IHN) [28]. A microarray analysis comparing wild and hatchery coho salmon was performed [29] to assess global effects on gene expression. Quantitative polymerase chain reactions (PCR) of the mRNA levels were carried out for three genes, including insulin-like growth factor I and II (IGF-I and -II), and growth hormone receptor (GHR) [30] to assess the expression of growth-hormone-related genes in naturally- and hatchery-reared fish.

### Swimming Endurance

The swimming endurance of both naturally- (n = 10) and hatchery-reared (n = 10) coho smolts was assessed in a swim tunnel on 25 and 26 June 2008. Each smolt was given 5 min to acclimate at 0.1 m · s^−1^, during which time its length was estimated using a ruler on the side of the swim tunnel and a calculation was done to estimate the rpm required for a velocity of 5 bl · s^−1^. After the acclimation period, the speed was increased to 5 bl · s^−1^ within 30 s. The time taken to reach exhaustion was recorded. A black cloth covered the up-flow part of the tank. Exhaustion was established when the fish stopped against the down-flow grate for 5 s. Following each trial, the fish were anesthetized, measured and, upon recovery, released with the non-experimental hatchery coho.

### Predator Avoidance

During 25 and 26 June 2008 two groups of naturally-reared (n = 20 in each) and two groups of hatchery-reared coho smolts (n = 20 in each) were placed in identical partially-covered troughs and allowed to acclimate for two days. The time to eat an egg dropped 30 cm from the edge of the covered area, was recorded five times for each of the four groups. After the fourth group was finished, a predatory attack was simulated on the first group by moving a plastic heron up and down in the tank quickly with its beak penetrating the water. The beak was kept just under the surface of the water as an egg was dropped into the tank. The time taken for the egg to be eaten was recorded. The heron's beak was removed after 90 s, and the trial was stopped after four minutes if the egg was still uneaten. Five repetitions were done per group per trial with a minimum of one hour between trials. Two trials were done per day, for a total of four trials.

### Migratory Behaviour

Sixty fish (n = 41 hatchery-reared, 19 naturally-reared) were tagged with 7 mm-diameter tags (VEMCO V7-2L-R64K transmitters, 7×18.5 mm, mass in air 1.4 g, mass in water 0.7 g, frequency 69 kHz, 60–180 s) according to methods in [31]. As the naturally-reared smolts were smaller, there were fewer that were large enough to tag (>11 cm), which is why the n values differed between groups. The surgeries were conducted on 22 and 23 June 2008 at the Chehalis River Hatchery. The mean fork length and mass of the hatchery-reared fish were 12.3±0.8 cm and 19.6±3.8 g, whereas the naturally-reared fish were 11.2±1.2 cm long and 13.9±4.4 g. The mean time spent in anesthetic was 5∶58±1∶21 min:s, in surgery 2∶21±0∶28 min:s, and in recovery 6∶03±1∶59 min:s. During the surgeries, the water temperature ranged from 8 −11°C and 10.4–11.6 ppm O_2_. The fish were released 24 hours later on the 23 and 24 June at nightfall (2130 h) from the hatchery together with 4,000 coho smolts. Manual tracking (VEMCO VR100) in the Chehalis River hatchery pool (C1, Fig. 1) was carried out for 24 h post-release.

Acoustic receiver arrays (VEMCO VR2s and VR3s) were moored along potential migratory routes to monitor the smolts' behaviour. Approximately 2 km downstream of the release site, one stationary receiver was located in the Chehalis River (C2, Fig. 1). Arrays were positioned in the Harrison River, both upstream and downstream from the entry point of the Chehalis River (H1, H2, Fig. 1) to monitor whether any tagged coho travelled upstream into Harrison Lake. Prior to joining the Fraser River, the Harrison River opens into a wide shallow area where coho smolts may residualise and exploit feeding opportunities (Fig. 1). A receiver array was positioned below this area, at the junction of the Harrison and Fraser Rivers (H3, Fig. 1), to determine whether coho were residualising in the Harrison River. Upon entering the Fraser River, the smolts had approximately 120 km to travel before entering salt water. Pacific Ocean Shelf Tracking project arrays located in the Fraser River, the Pitt River, and in the ocean monitored the further migrations (Fig. 1).

## Results

The wild and hatchery parent groups sampled in January 2007 did not differ in size when compared by origin and gender (Table S1). However, the analysis of control otoliths from the smolts in this study found distinct differences between fish reared in the natural and hatchery environments (Table S1). Whereas the naturally-reared smolts had otoliths that were small, dense, white and regular in shape with even growth rings, the otoliths from hatchery-reared fish tended to be large, thin, crystalline and irregular in shape, with a thick growth ring during the first winter. There was no observable difference in crystallization between the left and right otoliths. As hatchery-reared adults had a mean otolith degree of crystallization of 1.6 (40%), only those unclipped parents with otoliths less than or equal to 1 (25%) were considered to be of wild origin, and the others were removed from the study (the reason for only nine cross groups). Furthermore, wild-born females produced more eggs and more eggs surviving to hatch than hatchery-born females. Although the mean mass per egg from hatchery-born females was greater, the percentage of eggs surviving was similar for all cross groups (73%–99%; Table S1).

### Carrying Capacity

**Figure 3:**
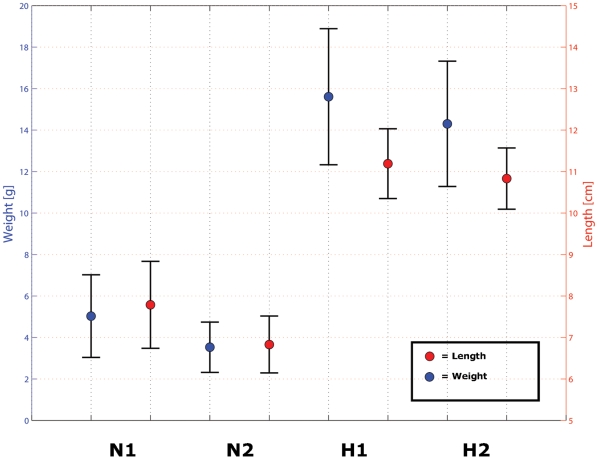
Length and weight of naturally-reared and hatchery-reared replicate groups. There was no difference between replicates; however the hatchery-reared fish were larger than the naturally-reared fish.

Growth comparisons between fish in the two natural and two hatchery rearing areas during February and April 2008 found no difference between replicates (February, lengths only: Hatchery 1, 10.6±0.9 cm; Hatchery 2, 10.5±0.6 cm); however, hatchery-reared fish were significantly larger than their naturally-reared siblings (April: Hatchery 1, 15.6±3.3 g, 11.2±0.8 cm; Hatchery 2, 14.3±3.0 g, 10.8±0.7 cm; Natural 1, 5.0±2.0 g, 7.8±1.0 cm; Natural 2, 3.6±1.2 g, 6.8±0.7 cm; P<0.001 for all, Mann-Whitney U test; Fig. 3). While it was possible that some of the naturally-reared smolts may have ingested bait from the minnow traps, this was not observed in later stomach content analyses. Near the end of the study there was a flood event that disturbed the barrier between the two natural habitat replicates, which made survival estimates between replicates not possible. The overall catchability of smolts in the natural rearing environment was 428 fish in 1,300 trap hours (0.33 fish per trap hour). Following that, very few fish were captured. Therefore, the approximate carrying capacity of the Chehalis River side-channel was 1.5 individuals per m, 0.3 individuals per m^2^, and 0.1 individuals per m^3^. The hatchery rearing density was 4,167 individuals per m^3^.

### Physiology

Size differences between genotype groups were negligible; however, differences in size, colouring, external damage, parasite levels, gill Na^+^/K+−ATPase activity and GHR mRNA levels were noted for rearing-environment groups (Table S1). The coho progeny could be assigned to families in eight out of the nine cross groups using microsatellite analysis. Within each cross group, maternal and rearing-environment effects on smolt size were compared if the sample sizes were large enough to detect an effect. In two of the hatchery-reared cross groups, no differences were found between pure wild, pure hatchery and hybrid genotype offspring in either mass or length (P = 0.8, P = 0.7, Mann-Whitney U test). However, two of the six pure-hatchery genotype full-sibling groups with large enough sample sizes (n = 15, 14, 14, 9, 8, 14) had a lower mean mass than the other four groups (P<0.04, Mann-Whitney U test). If data from their hybrid half-siblings were added to the analysis (same hatchery mothers, wild fathers; total n = 27, 21, 24, 9, 15, 24), there was no longer an observable difference in mass (P = 0.2, Mann-Whitney U test). Of the naturally-reared offspring, there was neither a difference in length nor mass between pure-hatchery-full-sibling groups, nor between pure-wild-full-sibling groups.

The parental cross groups were pooled by cross type (wild mother x wild father, wild mother x hatchery father, hatchery mother x wild father, hatchery mother x hatchery father) for analysis of body-size differences within each rearing environment. None of the genetic cross groups were significantly different in mass or length within a rearing environment (Table 1). However, there were differences in both length and mass between pooled naturally-reared (n = 124) and hatchery-reared (n = 255) smolts (mean ± stdev natural 8.5±6.3 cm, 5.4±1.7 g; hatchery 11.7±0.9 cm, 16.8±4.4 g; Mann-Whitney U test, P<0.05). The naturally-reared fish had higher variance than the hatchery-reared fish in fork length but not mass (F test, P<0.05). There was no clear trend in survival between cross groups in either the natural or hatchery rearing environments. In the natural environment, there were 31 pure wild offspring, 28 hybrids with wild-born mothers, 23 hybrids with hatchery-born mothers, and 42 pure hatchery offspring. In the hatchery environment there were 28 pure wild offspring, 96 hybrids with wild-born mothers, 52 hybrids with hatchery-born mothers, and 80 pure hatchery offspring.

**Table 1:** A comparison of growth (fork length and mass) between genetic cross groups (listed as mother x father, where W is wild-born and H is hatchery-born) and rearing environments during May and June 2008.

		Naturally-reared	Hatchery-reared
W×W	Length (cm)	7.8 (0.7)	11.5 (1.0)
	Mass (g)	5.1 (1.3)	16.2 (5.1)
	*N*	31	28
W×H	Length (cm)	7.9 (0.5)	11.6 (0.9)
	Mass (g)	5.3 (1.0)	16.8 (4.3)
	*N*	28	96
H×W	Length (cm)	8.1 (0.5)	11.5 (1.0)
	Mass (g)	5.6 (1.3)	15.9 (4.3)
	*N*	23	52
H×H	Length (cm)	8.0 (1.0)	11.8 (0.9)
	Mass (g)	5.8 (2.3)	17.5 (4.2)
	*N*	42	80

Whereas none of the genetic groups were significantly different within their rearing environment, all of the hatchery-reared fish were greater in length and mass than the wild-reared smolts (Mann-Whitney U test, P<0.05).

The hatchery-reared pre-smolts were lighter (more yellow) in colour than their naturally-reared siblings (green/brown with more pronounced parr marks). In the higher-density hatchery groups, the incidence of scale loss and fin damage was greater. The hatchery fish also had elevated amounts of adipose tissue and longer gill-rakers, whereas the naturally-reared fish had short gill-rakers and almost no body fat. Fifteen percent of the hatchery fish had eye damage, compared to only one percent of the naturally-reared fish. Skin parasites were observed in 37% of the naturally-reared fish and none of the hatchery-reared fish. Some of the naturally-reared fish also had parasites in their gills (7%) and body cavity (13%). No BKD or IHN was detected in either group. Gill Na^+^/K+−ATPase activitiy was higher in the hatchery-reared than the naturally-reared fish (1.56±0.35 µM ADP · mg protein^−1^ · h^−1^ and 1.29±0.33 µM ADP · mg protein^−1^ · h^−1^, respectively; P<0.02, Mann-Whitney U test).

The mRNA levels of IGF-I and -II were similar between rearing groups, but the mRNA levels of GHR were significantly lower in the hatchery-reared smolts than in their naturally-reared siblings (Table S1). Many genes exhibited greater than two-fold differences in mRNA levels between naturally- and hatchery-reared groups; however the differences were not statistically significant. The mRNA level of phosphoenolpyruvate carboxykinase (PEPCK) was significantly lower (P<0.05, Mann-Whitney U test) in the hatchery-reared fish than in the naturally-reared fish (Table 2). Two other genes with high log odds between rearing groups included tyrosine aminotransferase (TAT) and apolipoprotein B (apoB).

**Table 2:** The six genes that demonstrated the greatest log-fold differences in mRNA levels between naturally- and hatchery-reared smolts (significant difference noted by an asterix).

Rank	Gene	Accession No. of EST (UniGene ID)	Log fold change
1	Phosphoenolpyruvate carboxykinase 1, cytosolic (PEPCK)	CA053922 (Ssa.499)	−2.96* W>H
2	Unknown	CA060896 (Ssa.1836)	−2.10
3	Unknown	CB511331 (Ssa.24216)	−1.81
4	Tyrosine aminotransferase	CA056381	−1.44
5	Unknown	CA041582 (Ssa.6588)	1.53
6	Apolipoprotein B	CB511166 (Ssa.23489)	2.32

### Swimming Endurance

The swimming endurance of the naturally-reared fish was significantly greater than those reared in the hatchery troughs, even though they were smaller. The mean relative velocity swum (in bl · s^−1^) by the fish did not differ significantly between the naturally- and hatchery-reared groups (natural 5.0±0.5 bl · s^−1^, hatchery 4.8±0.3 bl · s^−1^; Mann-Whitney U test, P = 0.45). However, the naturally-reared fish took longer to fatigue (903±894 s) than the hatchery fish (207±146 s; P = 0.01, Mann-Whitney U test). The naturally-reared fish were also more agitated in the tunnel, scaring easily when their tails touched the down-flow grate. Conversely, the hatchery fish seemed to prop themselves up against the back grate and side of the tunnel with their tails intermittently.

### Predator Avoidance

**Figure 4:**
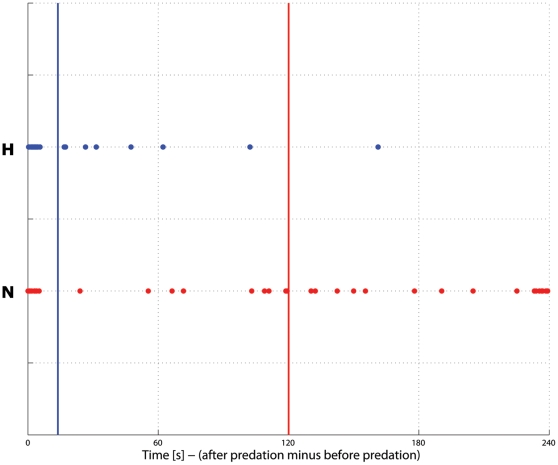
The recovery time following a predation event for naturally- and hatchery-reared coho salmon smolts, expressed as the difference between the time taken to eat before and after a predation event. Means are represented by blue (hatchery-reared) and red (naturally-reared) vertical lines.

The naturally-reared fish took longer to eat an egg placed in their tank both before and after a simulated predation than the hatchery-reared fish (natural: 2.3±2.7 s before, 122.3±94.2 s after predator, hatchery: 0.8±0.4 s before, 14.6±31.1 s after predator; P<0.001, Mann-Whitney U test). The naturally-reared fish also took longer to recover after the “predator attack” (Fig. 4; natural 120.1±93.0 s, hatchery 13.8±31.0 s; P<0.001, Mann-Whitney U test). Twelve of the 40 trials were stopped for the naturally-reared fish, as they had not eaten after four minutes, whereas no hatchery trial lasted longer than 162 s. The naturally-reared fish hid in a group beneath a covered area in the tank at all times when not feeding, whereas the hatchery fish were not as scared to venture out to the uncovered portion of their tank.

### Migratory Behaviour

Naturally-reared coho smolts were more likely to be detected downstream of the Harrison River than hatchery-reared smolts (natural: 26%, hatchery: 7%, Z value: 1.6, P<0.05, one-tailed Z-test). Of the 60 fish tagged, 83% were detected by moored receivers. All of the hatchery-reared smolts except two departed from the vicinity of the hatchery (C1, Fig. 1) the night after their release (mean 22±1 h post-release). The remaining two left the following evening. Two-thirds of the naturally-reared smolts began migrating during the night after their release and one-third stayed an extra night. It took the smolts approximately one day to travel from C1 to C2 (∼2 km), where some spent up to two days near the receiver (located in a deep pool in the Chehalis River). All of the fish arrived at the C2 and H2 arrays during the late night or early morning hours, which means that the smolts were likely travelling during darkness.

The four genetic cross types reared in the hatchery (n = 4 pure-hatchery, 1 pure-wild, 3 hybrids with wild-born mothers, 4 hybrids with hatchery-born mothers) had similar migratory behaviour and body size. All left the hatchery pool the night after release and travelled quickly downstream, arriving at C2 the same night. They arrived at H2 approximately three days post-release and spent very little time near the H2 array. Three of the smolts (1 pure hatchery genotype, 2 hybrids with hatchery mothers) were detected leaving the Harrison River. The two hybrids travelled quickly from H2 to H3, whereas the pure hatchery smolt spent two days in the shallow area of the Harrison River before travelling downstream to F1 and F2.

Most of the tagged fish stopped in the wide shallow area in the lower Harrison River (between H2 and H3). In total, only 8 of the 60 tagged fish (13%) were detected downstream of the Harrison River. Five of those eight were naturally-reared. The eight fish were similar in that they all travelled quickly downstream, spending 0–3 d between H2 and H3. One of the hatchery-reared fish was detected in the Fraser River at F1 and F2, five and seven days after its last detection at H3. Two of the naturally-reared fish were detected in the northern Strait of Georgia during July and August 2008 (NSOG, Fig. 1) and two were detected in Pitt River during October and November 2008 (P, Fig. 1).

Only one smolt (hatchery-reared) moved upstream and was detected at H1. This individual was recorded at H2 five days post-release, presumably heading downstream to the lower Harrison River. Two nights later, however, the fish was recorded again at H2, then at H1 the same night. It was never detected again and may have continued further upstream into Harrison Lake.

## Discussion

In the continuing debate of nature versus nurture, this study has found that the effects of rearing environment on phenotype and behaviour far outweighed the effects of any genetic differences existing between second-generation hatchery- and wild-born coho salmon in this system. Coho reared in a natural environment had more normal-type otoliths, greater swimming endurance and predator-avoidance abilities, and longer migrations than their hatchery-reared siblings. There were no observable differences in growth, survival or migratory behaviour between pure-wild- and pure-hatchery-genotype groups reared in the same environment. These results indicate that few, if any, genetic differences are present between the hatchery-reared and wild-born coho salmon studied. To what extent the wild-born fish are populated by previous generations of hatchery fish, however, is unknown.

The fact that there was little difference observed between the offspring of wild- and hatchery-born coho reared in the same environment suggests that there may not be a strong genetic difference between the two groups. While some studies have observed genetic influences on phenotypic differences between wild and hatchery populations [32]–[34], rearing environment seems to have a greater effect [35]. The lack of a noticeable genetic effect on the offspring of hatchery-born coho in the Chehalis River system could be because the wild-born individuals were actually the offspring of hatchery-reared fish—and one generation in the wild was not enough to restore a wild genotype. Or, perhaps there is such a small genetic effect from hatchery rearing that the difference between hatchery and wild genotypes is negligible. Testing for genetic differentiation between wild-born and hatchery-born coho in the Chehalis River may provide further clarity.

Studies examining the long-term captive breeding effects on coho populations in Washington and Oregon have found that the genotypes of many wild populations have transformed to more closely resemble the genotypes of hatchery-reared fish [36]. In some systems, the introduction of hatchery-influenced genes into wild populations by captive-breeding programs also reduced the fitness of those populations [5], [6], [19], [20]. This lack of an observable genetic difference between wild- and hatchery-born fish could thus be a cause for concern, especially if there is a risk that the accumulated genetic load of captive-breeding could contribute to a population's eventual extinction [37], [38].

Early rearing conditions seem to affect coho reproduction investment, which can directly influence overall fitness. Studies have found that hatchery-reared fish had greater ovary mass [39]; however, egg and ovary mass do not necessarily mean more surviving offspring. Hatchery-reared returning coho adults in the Chehalis River had fewer, but heavier eggs than wild-born coho from the same river. The total number of eggs surviving was greater from wild-born adults than from hatchery-born adults though, which may mean that the wild fish have greater reproductive success.

Balancing the risk of predation with the benefit of feeding is important for maximizing individual fitness, and is expected to be influenced by both genotype (e.g. inherent growth rate) and rearing environment (experience with predators in nature) [40]–[42]. The predator avoidance results demonstrate a major difference in the behaviour of naturally- and hatchery-reared coho smolts in these experiments. Fish with the tendency to be more careful around predators can avoid predation more easily and thus have greater survival. Predator-avoidance training in one study increased the in-stream survival of test subjects up to 26% over un-conditioned fish [43], [44]. The swimming performance of naturally-reared coho smolts was also noticeably stronger than that of their hatchery-reared siblings, which was consistent with reports in both freshwater and saltwater [45]. Other studies have found that post-release survival improved when hatchery-reared smolts were exercised [46]-[48]. Thus, a smolt's swimming and predator-avoidance abilities are important factors in their survival and overall fitness.

Feeding on natural sources as a pre-smolt likely increases a smolt's ability to find good quality food in the wild. Releases of hatchery-reared fish that had been hand-fed pellets were more surface-oriented and more likely to approach moving objects than were naturally-reared fish [49], [50]. Hatchery-reared fish supplemented with live feed had twice the foraging ability [51] and greater post-release survival [52] than those fed only pellets. The naturally-reared fish in this study were left to forage for naturally-occurring food sources. It is probable that they were able to find good quality food more easily than their hatchery-reared siblings. This ability may have been a factor in the longer migrations observed in the naturally-reared fish.

Naturally-reared coho smolts were more likely to be detected downstream of the Harrison River than their hatchery-reared siblings. Of the fish detected entering the Fraser River, 63% were naturally-reared, despite the fact that the naturally-reared fish made up only 32% of the total releases. These long migrants travelled quickly through the Harrison system and its shallow, predator-rich feeding areas. Many factors could have influenced this behaviour. The normal-type otoliths and better physical condition of naturally-reared smolts may improve their orientation, balance and swimming ability, allowing them to migrate further and faster. The foraging and predator-avoidance abilities of the naturally-reared smolts may have also increased their chances of survival.

The incidence of coho residualisation has not been fully investigated in the Fraser River system. This study has provided some evidence that coho may be residualising in the Harrison River and Harrison Lake. Mortalities or tag losses may have accounted for some of the fish that were not detected beyond the Harrison River. The possibility of some tagged fish passing by receiver arrays undetected also exists. Further quantification of coho residualisation in this system could be accomplished through the use of PIT tags and seining.

Distinguishing between hatchery-reared and naturally-reared salmon using only otoliths usually involves an analysis of early growth rings [53]. The otoliths of hatchery-reared coho adults returning to the Chehalis River had a high incidence of crystallization, however, which obliterated any growth rings. The degree of crystallization itself was a good determinant of which adults were of hatchery origin, and has been observed in other populations of coho in the Strait of Georgia [23]. The effects of abnormal otoliths on the behaviour and survival of salmon are unknown. However, they may account for some of the differences observed in migratory behaviour between hatchery and naturally-reared coho, and should be investigated.

The microarray analysis demonstrated that the metabolic rates of naturally- and hatchery-reared siblings differed. For example, the mRNA levels of cytosolic PEPCK gene were lower in the hatchery-reared smolts. PEPCK is a rate-limiting enzyme of gluconeogenesis [54]. PEPCK gene expression is controlled to maintain blood glucose level within homeostatic limits, and regulated by certain hormones including cortisol, glucagon and insulin [54]. Whereas cortisol and glucagon synergistically up-regulate gene expression, insulin is an inhibitor. The lower level of PEPCK gene expression in the hatchery-reared fish suggests that they have higher insulin levels than the naturally-reared fish. It remains unclear whether metabolic differences, such as the ones identified in this study, influence health and survival in salmonids. As metabolism can generally be controlled by limiting hatchery feed rations, further evaluation of the effects of metabolic differences on fitness would be useful to hatchery management.

The lower levels of Na^+^/K+−ATPase in the naturally-reared fish was inconsistent with the elevated levels of GHR mRNA in the same fish. The stimulatory effect of GH on smolting and ATPase would suggest that the elevated GHR mRNA reflects a more active role of GH in the naturally-reared fish—which should lead to more ATPase activity. Earlier work showed higher levels of Na^+^/K+−ATPase in wild than hatchery coho smolts from the Quinsam watershed [13], [16]. One possible explanation for the lower ATPase values in the naturally-reared fish is that they were already past the smolt window by the time of their sampling and release in June. If the smolts, particularly the naturally-reared smolts, were allowed to volitionally leave, they may have out-migrated earlier.

As the goal of salmon enhancement programs is to improve the conservation status and productivity of wild salmon stocks, smolt quality and fitness are of high priority. Behavioural deficiencies due to artificial rearing environments have been considered a primary cause of lower hatchery survival rates [55]. Some conservation-oriented hatcheries use enriched rearing environments, including matrices for egg and alevin development and in-stream structures and cover, to produce smolts that more closely resemble wild populations in both physiology and behaviour, while maximizing genetic diversity [56], [57]. Water temperature and quality is maintained to resemble local conditions, flow rates are higher to promote exercise, and food is introduced below the surface of the water using belt feeders [45], [58], [59]. Salmon raised in conservation hatcheries tend to have a more natural body colouring, better physical condition, lower disease rates and higher survival than traditionally-reared smolts [58]-[60]. Decreasing rearing densities improved smolt condition, growth, gill Na^+^/K+−ATPase activity levels, and survival [61], [62]. High rearing densities increased agonistic behaviour, which may effectively increase their risk of predation post-release [63], [64]. Volitional releases allow smolts to acclimate safely to the release environment and migrate out when they are physically ready. Releasing fish from in-river pens reduces stress, maintains out-migration diversity and allows smolts to travel at night when the risk of predation is lower [57], [65]. Conservation-oriented captive breeding programs may be one strategy to mitigate the effects of changing global conditions on salmon populations. However, with the possible long-term genetic effects arising from such strategies, some argue that the priority should lie with habitat restoration [66].

In the system studied, hatchery-reared fish are capable of returning to and spawning in the wild, and as such, wild-born fish may be comprised of a mixture of fish that have some hatchery ancestry and some ancestry from lineages that have been wild for many generations. Since the relative proportions of these ancestries in the population are unknown, it is not possible to determine whether the lack of observable genetic effects on offspring phenotype was due to 1) a significant mixture of their genotypes through interbreeding, 2) hatchery practices that maintain wild genetic diversity, or 3) strong selection for fish with appropriate “wild” genotypes (in natural marine environments for hatchery-reared smolts, or at all stages for wild-born fish with hatchery parents) that acts to canalize genetic variance for non-neutral loci between hatchery and wild-derived salmon in the population. However, if the Chehalis River hatchery is selecting for genotypes distinct from wild fish, and hatchery fish are contributing significantly to wild populations by breeding in the wild, then the data from the present study indicate that selection for wild genotypes in nature is occurring rapidly such that returning hatchery fish are not distinguishable phenotypically from those born of wild parents. Further studies tracking the contribution of hatchery genotypes to wild populations will be required to resolve these important questions.

## Supporting Information

File S1 Habitat assessment and carrying capacity.
(0.02 MB DOC)

Figure S1 A scale diagram of the natural rearing area near the Chehalis River Hatchery. The water flow begins at the circle on the right, next to the Chehalis Hatchery, flowing downstream to the left. The first habitat runs from the water source to the first fence. The second habitat is considerably shorter in length, from the first fence to the second fence. The numbers in pink indicate the width of channel in meters, and significant features are labeled.
(3.94 MB TIF)

Table S1 Phenotypic characteristics of naturally- and hatchery-reared coho salmon. Statistical differences were established at P<0.05 with the Mann-Whitney U test.
(0.09 MB DOC)
