# Supplementary material for: Genetic versus Rearing-Environment Effects on Phenotype: Hatchery and Natural Rearing Effects on Hatchery- and Wild-Born Coho Salmon
Source: PLoS One. 2010 Aug 19;5(8):e12261. doi: 10.1371/journal.pone.0012261 (PMC2924375; doi:10.1371/journal.pone.0012261)
Supplement: File S1 — Habitat assessment and carrying capacity. (0.02 MB DOC) [file pone.0012261.s001.doc]

**Habitat Assessment and Carrying Capacity**

To investigate the effects of stream length and area on the carrying capacity for coho, a habitat assessment of the natural rearing sites was carried out in late July 2007. Channel depth, bank height, width, substrate type, cover and vegetation type measurements were taken every 5 m of the stream’s length. An average channel depth was calculated from three measurements across the stream, at ¼, ½, and ¾ of the width. The surface area of each habitat was calculated as the product of its length and mean channel width. The cross-sectional area was the product of the mean depth and the width at each measurement point. The total stream volume was the product of its length and mean cross-sectional area. The flow velocity was the time taken for an object on the water’s surface to travel a distance of 10 m. This value was multiplied by a correction factor of 0.8 to account for the difference between mean water velocity and surface velocity. The total stream discharge was the product of the corrected velocity and the mean cross-sectional area of the stream at the same location where the flow measurements were taken. A benthic invertebrate survey was conducted 5 m from the downstream boundary of each rearing area in a 30 cm by 30 cm section of streambed. Water quality measurements (turbidity, pH, oxygen content and temperature) were also taken in the hatchery and natural rearing areas, and vegetation height was estimated using a clinometer.

The natural rearing channel replicates were similar in terms of temperature, pH, turbidity and percent cover, but differed in substrate and vegetation type. Both replicates had 66% cover, zero turbidity, temperatures ranging from 14 to15.5ºC and a pH of 6.6 to 6.9. However, while the upstream riparian zone was dominated by small shrubs and deciduous trees, the downstream portion was within a large coniferous forest. The upstream habitat also featured alternating sections of pool and riffle and many impediments to flow, including log jams, small waterfalls and a beaver dam (Fig. S1). Its substrate was variable, consisting of a mixture of cobble, silt, sand and woody debris. Downstream, the substrate (silt and woody debris) and flow pattern were more homogenous. The corrected average flow velocity 15 m downstream of the water source was 0.26 m s^-1^. When multiplied by an average cross sectional area of 0.57 m^2^ in this area, the flow rate was approximately 0.15 m^3^ s^-1^. The length and surface area of the upstream treatment was greater than those of the downstream segment (upstream: 240 m and 1,040 m^2^; downstream: 50 m and 435 m^2^). However, due to its greater mean width and depth, the downstream treatment had a larger average cross-sectional area and total volume (upstream: 1.12 m^2^ and 1,170m^3^; downstream: 4.74 m^2^ and 2,060 m^3^). The predominant taxon of benthic invertebrate was the caddis fly, *Cyrnus trimaculatus*, which is an indicator of good stream health [1]. The initial density in the natural-rearing areas was 19.7 fish per m^3^ in the upstream portion and 11.2 fish per m^3^ downstream.

**Reference**

1. Van der Geest HG, Greve GD, Kroon A, Kuijl S, Kraak MHS, et al. (2000) Sensitivity of characteristic riverine insects, the caddisfly *Cyrnus trimaculatus* and the mayfly *Ephoron virgo*, to copper and diazinon. Environmental Pollution 109(2): 177-182.
