# Supplementary figures and images for: Genetic versus Rearing-Environment Effects on Phenotype: Hatchery and Natural Rearing Effects on Hatchery- and Wild-Born Coho Salmon
Source: PLoS One. 2010 Aug 19;5(8):e12261. doi: 10.1371/journal.pone.0012261 (PMC2924375; doi:10.1371/journal.pone.0012261)

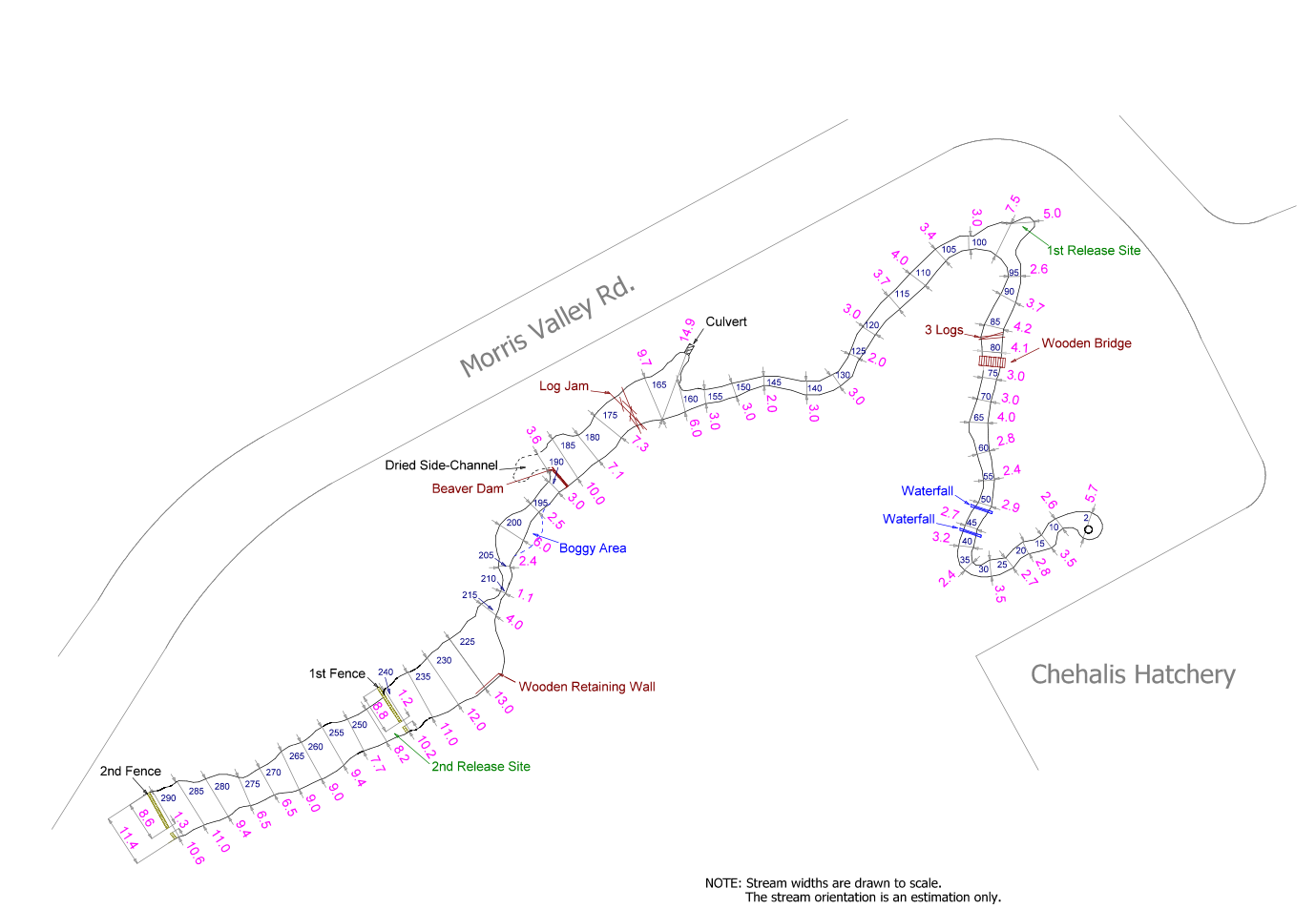

Supplement: Figure S1 — A scale diagram of the natural rearing area near the Chehalis River Hatchery. The water flow begins at the circle on the right, next to the Chehalis Hatchery, flowing downstream to the left. The first habitat runs from the water source to the first fence. The second habitat is considerably shorter in length, from the first fence to the second fence. The numbers in pink indicate the width of channel in meters, and significant features are labeled. (3.94 MB TIF) [file pone.0012261.s002.tif]
