# Supplementary material for: Genetic versus Rearing-Environment Effects on Phenotype: Hatchery and Natural Rearing Effects on Hatchery- and Wild-Born Coho Salmon
Source: PLoS One. 2010 Aug 19;5(8):e12261. doi: 10.1371/journal.pone.0012261 (PMC2924375; doi:10.1371/journal.pone.0012261)
Supplement: Table S1 — Phenotypic characteristics of naturally- and hatchery-reared coho salmon. Statistical differences were established at P<0.05 with the Mann-Whitney U test. (0.09 MB DOC) [file pone.0012261.s003.doc]

Table S1. Phenotypic characteristics of naturally- and hatchery-reared coho salmon. Statistical differences were established at P < 0.05 with the Mann-Whitney U test.

|  |  | Natural | Hatchery | Stats |
| --- | --- | --- | --- | --- |
| Mass, adult female (kg) | mean | 4.1 | 3.8 | N ~ H |
|  | stdev | 0.8 | 0.9 |  |
|  | *n* | 9 | 9 |  |
| Mass, adult male (kg) | mean | 3.6 | 4.1 | N ~ H |
|  | stdev | 0.8 | 1.0 |  |
|  | *n* | 9 | 9 |  |
| Length, adult female (cm) | mean | 70.6 | 69.3 | N ~ H |
|  | stdev | 4.6 | 4.4 |  |
|  | *n* | 9 | 9 |  |
| Length, adult male (cm) | mean | 69.5 | 71.2 | N ~ H |
|  | stdev | 4.7 | 6.0 |  |
|  | *n* | 9 | 9 |  |
| Mass / egg (g) | mean | 0.25 | 0.29 | N < H |
|  | stdev | 0.04 | 0.03 |  |
|  | *n* | 31,647 | 26,320 |  |
| N eggs / female | mean | 3,516 | 2,924 | N > H |
|  | stdev | 726 | 379 |  |
|  | *n* | 9 | 9 |  |
| N eggs hatch | mean | 3,285 | 2,818 | N > H |
|  | stdev | 763 | 351 |  |
|  | *n* | 9 | 9 |  |
| % eggs hatch | mean | 93.3 | 96.6 | N ~ H |
|  | stdev | 7.6 | 3.6 |  |
|  | *n* | 9 | 9 |  |
| Mass, smolt (g) | mean | 5.8 | 16.1 | N < H |
|  | stdev | 1.2 | 3.9 |  |
|  | *n* | 30 | 30 |  |
| Length, smolt (cm) | mean | 8.2 | 11.7 | N < H |
|  | stdev | 0.7 | 0.8 |  |
|  | *n* | 30 | 30 |  |
| Condition factor (g · cm^-3^) | mean | 1.04 | 1.00 | N ~ H |
|  | stdev | 0.11 | 0.11 |  |
|  | *n* | 30 | 30 |  |
| Otolith crystallization, adult (4 = completely crystalline) | mean | 0.1 (3%) | 1.6 (40%) | N < H |
|  | stdev | 0.5 | 1.5 |  |
|  | *n* | 34 | 34 |  |
| Otolith crystallization, smolt (4 = completely crystalline) | mean | 0.5 (13%) | 1.3 (33%) | N < H |
|  | stdev | 0.8 | 1.0 |  |
|  | *n* | 33 | 42 |  |
| Swimming time to fatigue (s) | mean | 831 | 207 | N > H |
|  | stdev | 920 | 146 |  |
|  | n | 10 | 10 |  |
| Time to eat pre-predation (s) | mean | 2.3 | 0.8 | N > H |
|  | stdev | 2.7 | 0.4 |  |
|  | *n* | 40 | 40 |  |
| Time to eat post-predation (s) | mean | 122.3 | 14.6 | N > H |
|  | stdev | 94.2 | 31.1 |  |
|  | *n* | 40 | 40 |  |
| Difference in time to eat  post- and pre-predation (s) | mean | 120.1 | 13.8 | N > H |
|  | stdev | 93.0 | 31.0 |  |
|  | *n* | 40 | 40 |  |
| Gill Na^+^/K+-ATPase  (µM ADP · mg protein^-1^ · h^-1^) | mean | 1.29 | 1.56 | N < H |
|  | stdev | 0.33 | 0.35 |  |
|  | *n* | 30 | 30 |  |
| Insulin-like growth factor I (IGF I) | mean | 1.0 | 1.1 | N ~ H |
|  | stdev | 0.4 | 0.8 |  |
|  | *n* | 30 | 30 |  |
| Insulin-like growth factor II (IGF II) | mean | 1.0 | 1.6 | N ~ H |
|  | stdev | 0.4 | 0.4 |  |
|  | n | 30 | 30 |  |
| Growth hormone receptor GHR | mean | 1.0 | 0.6 | N > H |
|  | stdev | 0.2 | 0.2 |  |
|  | n | 30 | 30 |  |
